# Workplace Bullying among Healthcare Professionals: A Quanti-Qualitative Survey in a Sample of Women Working in an Italian Healthcare Setting

**DOI:** 10.3390/ijerph20105859

**Published:** 2023-05-18

**Authors:** Daniela Acquadro Maran, Davide Minniti, Michele Presutti, Marta Alesina, Adelina Brizio, Paola Gatti

**Affiliations:** 1 Department of Psychology, Università di Torino, Via Verdi 10, 10124 Torino, Italy; 2 Azienda Sanitaria Locale Torino3, Collegno, 10093 Torino, Italy; 3 Department of Chemistry, Università di Torino, Via Pietro Giuria 7, 10125 Torino, Italy; 4 Department of Psychology, Università degli Studi di Milano-Bicocca, 20126 Milano, Italy

**Keywords:** workplace bullying, women, healthcare organization, well-being, mix method

## Abstract

The main objective of this study was to analyze, in a sample of female healthcare workers in Italy, the training needs to improve positive relationships in the healthcare organization. To better understand these needs, perceived workplace bullying and its consequences in terms of professional commitment and well-being were analyzed from a descriptive and quantitative perspective (or mixed-methods analysis). A questionnaire was completed online in a healthcare facility in northwestern Italy. The participants were 231 female employees. The quantitative data showed that, on average, the sampled population perceived a low burden of WPB. The majority of the sample expressed moderate engagement at work and moderate perception of psychological well-being. It is interesting to note that one element seemed to be overarching in the responses to the open-ended questions: communication, which emerged as a problematic element that affects the entire organization. The research data provide useful evidence for intervention in favor of an environment that helps to recognize the phenomenon and intervene in time, offering the possibility of accepting the discomfort and fatigue of healthcare workers and offering useful interventions to the individual and the team.

## 1. Introduction

The term workplace bullying (here in after, WPB) refers to violence by either one or more colleagues or superiors toward a victim who eventually develops a state of psychophysical discomfort as a result of the constant attacks and perceived injustices. The phenomenon is considered a form of aggression (psychological and sometimes physical [1]), which is used in the workplace, for example, with direct and hostile communication against a person who is unable or unwilling to defend himself. The victim thus becomes the target of oppressive and harmful behavior, gossip, innuendo, and public humiliation and is kept in this position of subjugation by frequent behaviors [2]. According to Khademi et al. [3], it can be considered as a violation of the human rights and dignity of the person, as it leads to impairment of the mental health and the physical and social development of the victim and also hinders professional development. Essentially, this form of abusive behavior involves the simultaneous presence of a co-worker who is victimized, targeted, and discriminated against and other co-workers who intentionally target the person and create a negative environment aimed at marginalizing, scorning, and tormenting them [4]. The individual consequences resulting from these episodes vary from depression and anxiety to negative somatic symptoms, stress, emotional exhaustion, fatigue, illness, decreased job satisfaction, and deterioration of mental health and well-being [5,6].

Many dysfunctional aspects of organizations and their corresponding degeneration are discussed extensively in the research already conducted on this phenomenon [7]. However, the issue of WPB appears to be more complex and difficult to address, as it is associated not only with organizational determinants but also with personal variables [8]. WPB results from a relationship between organizational factors (e.g., climate), work attitudes, and health [9]. Einarsen et al. [10] (see also [11]) divide WPB into three distinct categories: work-related bullying, which consists of withholding relevant information from the victim or setting unreasonable work deadlines; personal bullying, which consists of spreading rumors and gossip about the victim; and physical bullying, which consists of threats of physical aggression or abusive behavior such as raising of the voice against the victim (the latter is rarely practiced in the workplace; see Einarsen et al. [6]). In determining WPB experiences, the influence and importance of the work environment and its relationship with the individual is evident. According to Valle et al. [12], WPB is considered a symptom of organizational dysfunction. Empirical research has shown that it correlates with many characteristics of the work environment, including organizational problems, experienced role conflict, work control, high workload, increasing haste at work, high stress, organizational restructuring, management changes, low satisfaction with leadership or management styles, the social or organizational climate, unsatisfactory relationships in the workplace, conflict in general in the work unit, and difficulty talking about problems in the work group [13,14,15]. An organization characterized by WPB is particularly stressful and activates the process of health impairment, leading to increased health problems such as decreased well-being.

### 1.1. Prevalence and Consequence of WPB in Healthcare Sector

According to the Fifth European Working Conditions Survey (Eurofound, [16]), the prevalence rate of workplace bullying was 11.3% among healthcare workers. Females and people under 30 are those most affected by this phenomenon. A previous study published by the World Health Organization found that health professionals had been insulted 39.5% of the time in Brazil, 32.2% in Bulgaria, 52% in South Africa, 47.7% in Thailand, 27.4% in Portugal, 40.9% in Lebanon, and 67% in Australia in one year [17]. Furthermore, Spector et al. [18] conducted a quantitative review of 136 healthcare studies. They reported that the prevalence of WPB ranged from 57.6% in hospitals to 67.7% in psychiatric facilities. The average percentage of perceived WPB also varied between geographic regions: the Middle East (86.5%), England (39.5%), Asia (29.8%), and Europe (8.8%). The highest rate of non-physical peer violence occurred among nurses in Asia (50.2%), followed by the Middle East (44.9%), Anglo-American countries (USA, Canada, UK, and Australia) (37.4%), and Europe (27.6%). Asian, Anglo-American, and Middle Eastern nurses were similarly likely to experience physical violence, at 7.3%, 6.6%, and 6.0%, respectively (see also [19]).

Even before studies on WPB were conducted, research on workplace stress showed that poor relationships with colleagues and supervisors were associated with lower job involvement and well-being [20]. There is growing evidence that WPB can increase the risk of errors, result in poorer patient care, and might represent a threat to safety (see for example [21,22,23]). WPB can act as a disruptive behavior that undermines teamwork and the ability to develop a culture of safety [24]. Studies have also shown that WPB leads to the suppression of discussion and help-seeking behavior [25,26], which impairs individual performance, teamwork, and communication [27]. When negative relationships were measured by asking respondents how often they were criticized and harassed by colleagues and supervisors, they were found to be strongly associated with both global work stress and negative feelings about work. Negative relationships with colleagues were also associated with depression and negative relationships with supervisors with overall physical health [28].

Moreover, WPB is considered a serious social stressor in the workplace [29] and a critical life event [30] that affects well-being. In the Job Demands–Resources (JD-R) model of work stress [31], WPB is considered as job demands that contribute to stress by depleting energy reserves. Job demands are physical, social, or organizational aspects of work that require sustained physical, psychological, cognitive, or emotional effort [32]. The organization characterized by WPB is particularly stressful and activates the process of health impairment, leading to increased health problems such as decreased well-being and an increase in psychological health symptoms [32]. Acquadro Maran et al. [33] found that the consequences of WPB were also relayed to those who were witnesses of the phenomenon and perceived a higher severity level of mental health problems in this group than among non-witnesses. Overall, as suggested by Haldorai et al. [34], WPB affects a workers’ feeling of connection or engagement with his or her job as a responsibility assigned to him or her. Job involvement is the extent to which an employee identifies with his or her job and is the way to measure the extent of a positive relationship within the organization. Therefore, it is very important for employees in organizations who see meaning in their work to have an impact on the results of their work. Job involvement is expected to reduce work stress and allow employees to enjoy their work without feeling that they are being heavily burdened by employee engagement that occurs in response to a particular task or role in a work situation. In other words, the type of work or work environment affects who is engaged or disengaged in their work. In the case of WPB, as described by Mugiono et al. [35], the person in the work relationship is not influenced only by the work itself.

### 1.2. Current Study

Previous studies conducted in Italy have found a higher prevalence of WPB compared to Northern European countries [36,37]. The Italian Association for Human Resources Management [38] conducted a survey of 600 HR managers in 2022. The survey shows that 40% of the victims are women. In addition, Italian health and safety worker representatives who participated in the ESENER survey reported that they received 15.1% calls to take action against WPB in 2006–2009. The review study by Civilotti et al. [39] showed that the type of WPB that Italian healthcare workers experience most often, similar to their counterparts around the world, consists mainly of verbal aggression and threats (11.9 to 93.3% of healthcare workers reported being victims of this form of violence). Previously, the study by Adib et al. [40] showed that young and inexperienced employees are more vulnerable to attacks, while greater seniority and even age itself seem to be protective factors. In addition, Ferri et al. [41] showed that, among 745 healthcare workers in a general hospital in Northern Italy, women were particularly affected by workplace aggression (see also [42]). This finding was confirmed by Ielapi et al. [43] and La Torre et al. [44]. Data from the study by Ielapy et al. [45] showed that female gender was associated with a 2.6-fold higher risk for the presence of aggression among healthcare workers. Among 3129 Italian healthcare workers, La Torre et al. [44] found that women were significantly more likely to experience bullying (16.4% vs. 12.3%) than men. Taken together, the results show that it is necessary to study the phenomenon among women, as they are particularly affected. Understanding the characteristics of victimization can help organizations to intervene with methods and tools tailored to the nature of the target group. As Ielapi et al. [43] note, a major difficulty in adopting intervention measures related to workplace violence is the opacity of definition and arbitrariness in assessment by victims, perpetrators, and institutions, leading to difficulty in recognizing WPB and/or fear of reporting episodes of violence: these are the most common causes of underreporting [45,46,47]. Therefore, it is important that staff training promotes norms and values that foster a culture that supports positive relationships and avoids misconduct that leads to WPB.

Starting from these premises, the main objective of this study was to analyze in a sample of female Italian healthcare workers the training needs to improve positive relationships in the healthcare organization. To better understand their needs, the perceived WPB and its consequences, in terms of job involvement and well-being, were analyzed from a descriptive and quanti-qualitative perspective (or mixed-method analysis). This analysis also allows us to capture the respondents’ point of view without pre-determining their answers. This approach is widely used in social science research and has been used to study the perceptions of physical and verbal aggression by healthcare workers. It has also been used, for example, to examine workers’ descriptions of violent behaviors and perceptions of the safety climate in the organization [48].

To the best of our knowledge, this is the first study that aims to analyze the training needs in a sample of female healthcare workers for the prevention of WPB from a quanti-qualitative perspective. Based on the above literature, the hypothesis of this study is that healthcare workers who report higher levels of WPB will have lower levels of job involvement and perceived well-being. We do not have specific hypotheses about the relationship between WBP, professional engagement, perceived well-being, and the lexical terms used to indicate training needs. Therefore, we intend to analyze this relationship from an exploratory perspective.

## 2. Materials and Methods

An anonymous online questionnaire, specifically designed for the purpose of this study, was distributed to the sample. In the first part of the questionnaire, the sociodemographic variables were assessed (age, work experience, organizational role).

To assess the nature of WPB experienced by participants, the Negative Acts Questionnaire—Revised (NAQ-R) [5] was used in the Italian version [49,50].

The items followed an operational approach in which participants were asked to indicate how often they experienced different potential WPB behaviors. WPB referred to two types of misconduct: personal bullying, i.e., hostile actions toward an individual (PB; e.g., “spreading gossip and rumors about your colleague/supervisor”) and work-related bullying, i.e., behaviors related to the work of the individual who is the target of the bullying (WRB; e.g., “someone withholds information that affects the performance of your colleague/supervisor”). It should be noted that the items never use terms directly related to concepts such as abusive behavior or bullying. All items were rated on a 5-point Likert scale (1 = never, 5 = every day). In this study, the PB Cronbach’s alpha = 0.95 and the WRB Cronbach’s alpha = 0.80.

The Majer–D’Amato Organizational Questionnaire (M-DOQ, [51]) was used to assess job involvement. The subscale analyzing job and organizational involvement (M-DOQ_JI) was used (5 items, α = 0.74, e.g., “My job is thrilling and exciting”). The questionnaire was developed to measure organizational climate as a multidimensional construct. The instrument consists of 70 items proposed in the form of statements to which respondents express their agreement on a 5-point Likert scale (1—completely false, 5—completely true).

The Italian short version of the Psychological General Well-Being Index—A (PGWBI-A) [52] was used to assess perceived well-being. The six questions cover the following domains: Anxiety, Depression, Positive Well-Being, Self-Control, General Health, and Vitality (α = 0.85, example item “I felt cheerful, lighthearted during the past four weeks”). An increasing score, ranging from 0 to 30, or higher scores indicate better psychological well-being.

In the last part of the questionnaire, the participants were asked to make suggestions for improving the workplace from the point of view of the relationship.

### 2.1. Procedure

The ethics committee of the University of Turin approved this research project (No. 0654311—7 December 2021). After the meeting with the managers of the healthcare organization, an internal memo was sent to female employees. The memo included a description of the project and the link to the questionnaire that was active during the period from December 2021 to May 2022. All participants were informed that they could leave the questionnaire at any time and that their responses were anonymous. In addition, participants were informed that they could choose not to answer the question if they felt bothered by it and that they could contact free services offering psychological support if they had negative feelings. This study was conducted in accordance with data-protection regulations. In accordance with the Declaration of Helsinki [53], an information letter and a consent form were given to the participants along with the questionnaire. It took about 20 min to complete the questionnaire. There were no grades, credits, or money for this activity, so it was voluntary.

### 2.2. Participants

The health facility is “ASL TO3”, located in the northwest of Italy. This health facility has 4019 employees, including health, administrative, and support staff. Of these, 3040 were female (75.64% of the total number of employees). This health facility is the reference point for the health care of 581,281 people. Before conducting the survey, we calculated the sample size. Based on the consideration that the total number of female employees in the health facility was 3040 and that we did not know a priori the percentage of people who had suffered violence, we assumed a prevalence of 16.4%, in agreement with La Torre et al. [44]. The minimum number of observations required to achieve a 95% confidence level and a 5% margin of error was 194, and the 231 responses we collected allowed a sufficient margin. The calculations were performed using Calculator.net (https://www.calculator.net/sample-size-calculator.html?type=1&cl=95&ci=5&pp=26&ps=311&x=123&y=18 (accessed on 15 June 2022). The 231 participants were 47.9 years old on average (SD 10.9) and had worked in this healthcare setting for 18.3 years on average (SD 12.7). Moreover, 65.5% (*n* = 150) were married or in a committed relationship, 20.1% (46) were single, 11.8% (27) were divorced, and 2.6% (6) were widowed. Furthermore, 66.1% (152) had children, while 33.9% (78) did not. Of the 152 women who had children, 72 had minor children. Meanwhile 64.3% (*n* = 148) of respondents were health staff, while 35.7% (*n* = 82) were administrative and support staff. In all, 89.1% (*n* = 205) had a permanent contract and 10.9% (*n* = 25) had a temporary contract.

### 2.3. Data Analysis

Quantitative data were processed with SPSS version 28 (IBM Corp., Armonk, NY, USA). The reliability of the measures was assessed with Cronbach’s alpha. The qualitative data from the questionnaire were analyzed using Alceste 2018 [54,55]. As De Alba [56] argues, this program is an adequate analytical tool for the study of discourse. The author emphasizes that this program is not only a method for analyzing textual data that contributes to the understanding of the structure and organization of discourse but also allows for the highlighting of relationships between lexical worlds that would be difficult to find using traditional methods of content analysis. Alceste processes verbal data according to a descending hierarchical classification: the text is first divided into elementary contextual units (u.c.e.) and then into homogeneous classes. Homogeneity is based on the idea that a given topic is expressed by similar words. The software uses symbols to indicate the type of root in the words. If the word is followed by the symbol +, it means that only the root of the word is recognized (e.g., manager, management). Throughout the software, it is possible to isolate and separate internal classes within specific populations: each class is formed based on the common occurrence of elementary contextual units. The software identifies the classes that are most homogeneous in content; thus, the classes have a semantic lexical universe that is distinct from the others. The software also performs a χ^2^ test to identify the association between the words that make up the classes. This process allows the identification of the specific vocabulary for each class, i.e., the lexical worlds in the text [57], and the words that occur once (called hapaxes). The hapax highlights the number of unique words, i.e., the terms that do not occur in the specific sample (the hapax is not included in the output of the classes). In addition, the software offers the possibility to analyze the classification of the class tree (dendrogram); it is possible to slide from the lexical worlds to thematic reference universes. The software also allows us to insert an illustrative variable to anchor the text. Together with the text, these variables are thus recognized by the software, which determines their anchoring with the analyzed text: they allow us to identify the specific features of the semantic universe of the characters. For the present study, we created a corpus of text containing the descriptions of the training needs described by the participants. The illustrative variables included in the corpus were NAQ_PB, NAQ_WR, M-DOQ_JI, PGWBI-A, work experience, and role.

## 3. Results

Regarding the demographic variables related to work experience, 73.9% of the sample (i.e., 170 respondents) had a full-time job and 89.1% (205) had a permanent contract. Furthermore, 64.3% (148) of respondents worked in wards, 24.8% (57) in administration, and 10.9% (25) in other areas (management, for example). The role was classified based on Italian law [58]. The work categories are listed in Table 1.

On average, participants had a work experience of 18.26 years (range ≤ 1–40 years, s.d. = 12.73 years). The years of work experience were divided into three categories. For the purpose of this study, the range 0 to 9.5 years was indicated with the number 1; 33.3% of the participants indicated this length of service. The range 10–25 years was reported as number 2 (35.1%) and the range 26–40 years was reported as number 3 (31.6%).

As shown in Table 2, most of the sample had a stable relationship. In addition, most reported having at least one child. The most frequently reported contract type was full-time, and the majority of respondents indicated a permanent contract. Regarding the field of work, most of the participants indicated that they worked in a hospital ward.

### Quanti-Qualitative Analysis

As shown in Table 3, regarding personal bullying, most respondents reported a percentage of perception of the phenomenon that we classified as low. Note that we established thresholds that we believe are useful to better understand the extent of exposure to WPB. This assessment strategy was used in a previous study and allowed us to identify exposure to the phenomenon among victims of WPB and the consequences from an emotional distress perspective. Regarding exposure to work-related bullying, the data again suggested that participants rated the exposure as low. Most participants also reported moderate levels of job engagement, and the majority of participants reported moderate levels of well-being.

**Figure 1:**
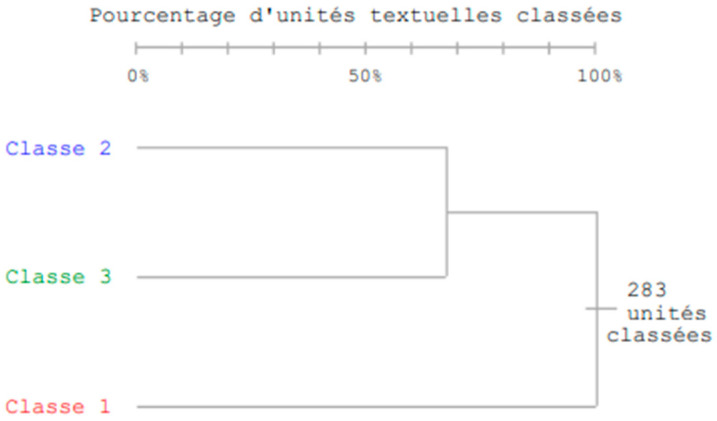
Dendrogram of descending hierarchical classification.

The analyzed corpus consisted of 328 u.c.e., with a total of 5103 forms in the text; the average frequency was 4 per form. The number of hapaxes, i.e., words used only once, was 862. Since 87% of the units were classified, the power of the analysis was considered very high. Based on the co-occurrences between forms and elementary context units, the corpus was divided into three classes using a hierarchical descending classification, the dendrogram of which is shown in Figure 1.

As shown in Figure 1, the classification procedure opposed classes II and III versus I. Classes II (91 units) and III (103 units) explained 68.55% of the variance. Class I contained 89 units and explained 31% of the variance. For each class, the first five words that characterize the class are identified in order of chi-squared in Table 4 with illustrative variables. The illustrative variables that characterize the class are inserted for each class. For example, if a class has the illustrative variable NAQ_WR_HIGH_, it means that the participants who used this type of language had a higher score on the NAQ_WR scale than the respondents in the other classes.

In Class II, reference was made to the need for training that focuses on the specific training course for each service, the ability to work as a team, and knowledge of the procedures to be used. It is interesting to note that this group of words was used by participants with a low perception of negative actions from the point of view of relationships at work, with high job involvement, and with high psychological well-being. There are no sociodemographic variables associated with this class, so it is transversal in terms of work experience and role. Below are examples of sentences from the questionnaires:

“My group lacks digital skills, we need courses on team building and teamwork. Also, communication effectiveness should be worked on”.

(role: D; work experience: 1)

“Specific professional development courses are required for operators in the various departments”.

(role: D; work experience: 3)

In Class III, the words used to reply to the question referred to the management of groups, to the group dynamics, to emotion and conflict. This group of words was used by participants with a medium and high perception of negative actions from the point of view of relationships at work, with a medium job involvement and those who had a low role in the organization. Below is an example from the questionnaire:

“We do not know how to communicate and how to manage relationships and groups. Some coordination figures create stress and internal separation of teams instead of creating unity and collaboration”.

(role: C; work experience: 3)

Class I collected the words used by those with the lowest organizational role and those who indicated the highest score in work-related negative acts and the lowest job involvement. The words referred to difficulties in the relationship with colleagues, lack of personnel, and fatigue:

“There is a lack of confrontation with supervisors, exchange with colleagues, it is not organic in programming, we always work in emergency and with few tools and personnel. We need calm to have a clear idea of the tasks”.

(role: D; work experience: 2)

“This is a difficult task that I and my colleagues always try, considering that after two years (of pandemic) we often find ourselves without material and human resources”.

(role: D; work experience: 1)

## 4. Discussion

Data from this study may help to better understand the training needs of women working in the health sector and living in an environment characterized by WPB. The quantitative data show that, on average, the studied population perceived a low burden of WPB. Most of the sample expressed moderate engagement at work and moderate perceptions of psychological well-being. The most interesting results were concerning the qualitative part: the answers to the open questions of the questionnaire.

It is interesting to note that there is one element that seems to be overarching in the open-questions’ answers: it is communication, which turns out to be a problematic element that affects the entire organization. As described by Leymann [59], WPB finds fertile ground in an environment characterized by poor interpersonal relations that manifest themselves in hostile communication. Therefore, the need to intervene in the modalities and content of communication might make it possible to reflect on relationships, group dynamics, the ability to coordinate teams, clarify procedures, and clarify organizational roles. The expressed need for more and better communication fits a profession that has been severely fatigued by the pandemic period. This time has led to overload in the healthcare system, where staff are exhausted and have few personal resources to address new organizational challenges. As Barello et al. [60] notes, work demands have increased due to the successes of the past two years, to which new demands such as vaccination campaigns have been added. Given these organizational demands, it is important that organizations provide resources, such as increasing staffing, facilitating recovery, and providing services to support health workers. Overtiredness of healthcare workers in a WPB-driven environment can put healthcare workers at risk for exhaustion or burnout, which affects mental and physical health and staff performance [61].

At a management level, Daniel [62] suggested some strategies to prevent WPB that include (a) enforcing a low tolerance threshold for bullying through laws similar to those for sexual harassment or by enforcing internal rules, (b) training all employees in organizations to know what constitutes workplace bullying and the penalties for violating laws or rules, and (c) standardizing professionalism as a necessary component of workplace behavior through role models. Cleary et al. [63] proposed a zero-tolerance approach based on the belief that organizational toleration of WPB is a necessary condition for its occurrence. Previously, Hesketh et al. [64] argued for a “broken windows” approach, similar to the justice system, in which penalties for minor offenses are increased in the belief that lowering the threshold for censure will prevent more serious offenses. To effectively apply the zero-tolerance policy, it must be written and easily accessible, and procedures must be developed for its implementation so that employees and managers adhere to it. Supporting managers through training and other professional development opportunities is an important consideration. However, as van Heugten [65] noted, a zero-tolerance approach may ignore the complexity of interpersonal and organizational relationships in which WPB can be better seen as a symptom of dysfunctionality rather than the cause. Other useful interventions are external mediation between the parties involved in the WPB and an education course about the concept of WPB. Most importantly, as Pariona-Cabrera et al. [66] point out, access to social resources, including social rather than individual support, could be of great benefit. This reduces the tendency to withdraw and lose confidence and may provide the best opportunity to successfully address inappropriate behavior in the workplace. Achieving higher levels of social support and lowering the tolerance threshold for WPB requires a better understanding of the bystander phenomenon as it relates to the situation. Building healthy workplaces requires consulting with workers and incorporating their input into decision making to give workers more control over their work [67]. Policies and practices should promote understanding and consideration of the diverse needs of people in the workplace, including those responsible for teamwork.

This study has several limitations. First, it is a cross-sectional study, so the results can only refer to the sample included in this study. The results may not be generalizable. Longitudinal studies and the inclusion of male workers could be useful to better understand the phenomenon of WPB and the methods of prevention and intervention. In addition, further research over a longer period is desirable to understand the extent to which efforts during the pandemic may have influenced participant responses. The possibility of conducting focus groups with participants who were more likely to have been exposed to negative actions in an occupational context could also provide further useful information for prevention and intervention. In addition, we did not consider some sociodemographic variables. Working in a particular sector or educational level could be useful information to better understand what type of organizational intervention can be proposed. Further research could consider these variables as part of the qualitative research. Another limitation is the potential for bias in participation in this study. This refers specifically to social desirability [68], meaning that participants may have answered the questions in the questionnaire in a way that showed they agreed with the research objective. Indeed, in this type of research, it is important to consider the stigmatization of the phenomenon: victims tend not to tell how they became victims but to refer to a hostile environment. Further research might consider using a scale to examine the propensity for social desirability and the level of concern about organizational stigma, which can lead to unreported WPB and victims not seeking support. Another limitation is the statistical method used. Alceste allows analysis of the lexicon used by certain subgroups. Other statistical methods, such as latent class analysis, allow the identification of qualitatively different subgroups within populations that share certain characteristics [69]. Future research could use this method to detect latent (or unobserved) heterogeneity in samples.

## 5. Conclusions

The research data provide useful evidence for intervention in favor of an environment that helps to recognize the phenomenon and intervene in a timely manner, providing the opportunity to welcome the discomfort and fatigue of healthcare workers and offer useful interventions to the individual and the team.

## Figures and Tables

**Table 1:** Participants’ work categories (*n* = 231). Data in percentage.

Categories	Description	%
B	Degree of autonomy: execution of tasks based on established procedures; degree of responsibility: related to the proper execution of procedures; required qualification for access: compulsory education plus any professional qualification	12.5
C	Degree of autonomy: execution of tasks related to procedures with varying degrees of difficulty based on partially established criteria; degree of responsibility: in relation to the general correctness of the procedures managed; qualification required for access: secondary school diploma	13
D	Degree of autonomy: execution of tasks related to different, non-predetermined solutions; degree of responsibility: related to the technical and/or administrative correctness of the chosen solutions; qualification required for access: college degree	64.5
H	Head of apical structure	3.9
M	Manager	6.1

**Table 2:** Participants’ sociodemographic characteristics (*n* = 231). Data in percentage.

Variable	Options/Analyses	%
Civil Status	Married/in a stable relationship Single Divorced/separated Widowed	65.5 20.1 11.8 2.6
Having Children	Yes No	66.1 33.9
Type of Contract—Hours Worked	Full-time job Part-time job/work shifts	73.9 26.1
Type of Contract—Duration	Permanent contract Temporary contract	89.1 10.9
Working Area	Hospital ward Administrative staff Other roles	64.3 24.8 10.9
Age	Mean SD	47.93 years 10.918

Note: Considering the very low number of missing values in the sample (i.e., 1 or 2), we chose to use valid percentages.

**Table 3:** NAQ_PB, NAQ_WR, MOQ_IJ, PGWI-A. Means, standard deviation, cutoffs, frequencies, and percentage (*n* = 231).

Variable	M (SD)	Cutoff Values	N	%
NAQ_PB	1.466 (0.730)	1 = Low = 1.000–2.099 2 = Moderate = 2.100–3.509 3 = High = 3.510–5.000	201 23 6	87.4 10.0 2.6
NAQ_WR	1.936 (0.847)	1 = Low = 1.000–2.099 2 = Moderate = 2.100–3.509 3 = High = 3.510–5.000	161 54 16	69.7 23.4 6.9
M-DOQ_JI	3.212 (0.881)	1 = Low = 1.000– 2.999 2 = Moderate = 3.000–3.999 3 = High = 4.000–5.000	71 105 55	30.7 45.5 23.8
PGWBI-A	4.109 (0.822)	1 = Low = 1.000–3.500 2 = Moderate = 3.501–4.500 3 = High = 4.501–5.834	57 106 68	24.7 45.9 29.4

Note: NAQ_PB = Negative Act Questionnaire—Personal Bullying; NAQ_WR = Negative Act Questionnaire—Work-Related Bullying; M-DOQ_JI = Majer–D’Amato Questionnaire—Job Involvement; PGWBI-A = Psychological General Well-Being Index.

**Table 4:** First five words characterizing Classes I, II, and III.

Class I		Class II		Class III	
Words	χ^2^	Words	χ^2^	Words	χ^2^
Difficult+	80	Profess+	58	Manag+	83
Colleague+	51	Training	40	Dynamic	60
Relation+	41	Team	25	Group+	58
Fatigue	39	Work	18	Emotion+	16
Lack	37	Procedur+	14	Coordinat+	14
Illustrative variables: NAQ_WR_HIGH,_ M-DOQ_JI_LOW_, Role_B		Illustrative variables: NAQ_PR_LOW_, M-DOQ_ JI_HIGH_, PGWBI-A_HIGH_		Illustrative variables: NAQ_WB_MODERATE_, NAQ_WR_HIGH_, M-DOQ_JI_MODERATE_, M-DOQ_JI_HIGH_, Role_C.	

Note: NAQ_PB = Negative Act Questionnaire—Personal Bullying; NAQ_WR = Negative Act Questionnaire—Work-Related Bullying; M-DOQ_JI = Majer–D’Amato Questionnaire Job Involvement; PGWBI-A = Psychological General Well-Being Index.

## Data Availability

The datasets generated for this study are available on request to the corresponding author.
